# The Fire and Explosion Hazard of Coloured Powders Used during the Holi Festival

**DOI:** 10.3390/ijerph182111090

**Published:** 2021-10-21

**Authors:** Bożena Kukfisz, Robert Piec

**Affiliations:** 1 Main School of Fire Service, Faculty of Security Engineering and Civil Protection, 52/54 Słowackiego Street, 01-629 Warsaw, Poland; 2 Main School of Fire Service, International Security Institute, 52/54 Słowackiego Street, 01-629 Warsaw, Poland; rpiec@sgsp.edu.pl

**Keywords:** fire safety, air pollution, Holi dust

## Abstract

During the world-famous Holi festival, people throw and smear each other with a colored powder (Holi color, Holi powder, Gulal powder). Until now, adverse health and environmental effects (skin and eye irritation, air pollution, and respiratory problems) have been described in the available literature. However, the literature lacks data on the flammable and explosive properties of these powders during mass events, despite the fact that burns, fires, and explosions during the Holi festival have taken place many times. The aim of the article is to present the fire and explosion parameters of three currently used Holi dust and cornflour dust types as reference dust. The minimum ignition temperature of the dust layer and dust cloud, the maximum explosion pressure and its maximum rate of growth over time, the lower explosion limit, the limit of oxygen concentration, and the minimum ignition energy were determined. Tests confirmed that the currently available Holi powders should be classified as flammable dusts and low-explosive dusts. The likelihood of a fire or explosion during mass incidents involving a Holi dust-air mixture is high.

## 1. Introduction

Holi is one of the major festivals of India and is celebrated with enthusiasm and gaiety every year. It is a festival arising from cultural and religious practices and has recently been gaining popularity in diverse countries of the world. The festival starts one day after the full moon in the Hindu month of Phalguna, which falls in the middle of March. It is widely known as the spring festival in the eastern Indian state of West Bengal, as well as in the area that surrounds the city of Kolkata. Initially, Holi was an agricultural festival of fertility and harvest to welcome the spring, but it also celebrates certain aspects of Hindu mythology and legend. Early on, bright flowers blossoming in the spring period were used as raw materials to make the different shades of the Holi colors. According to popular belief, most of these plants had medicinal properties that were advantageous to the skin.

The festival is a free-for-all of colors, where large numbers of people gather in open streets and parks to throw colored powders at each other, listen and dance to music, and share special Holi food and drink delicacies. This ancient Hindu religious festival has become popular among non-Hindu populations and people of other communities outside Asia, and similar events are also held across the United Kingdom [1]. The Holi traditions have also been transferred to Europe and adapted to become a commercial party event. Color runs are currently gaining in popularity. These are non-traditional road races organized to celebrate health, joy, and solidarity among participants. Partakers put on brightly colored t-shirts, some of which are color-powdered. As runners pass by, the crowds of spectators also throw powder at them. These powders have been primarily engineered from a comparable, but possibly dangerous formula, related to those that are used during the Holi festival itself.

Studies have shown that cultural and religious practices may considerably affect the health and safety of people or the environment. Indeed, it has been found that certain practices often give rise to health hazards. Unfortunately, due to the spread of industrialization and urbanization, natural colors have been substituted through the years by low-priced industrial dyes, which are generated in chemical processes.

Many scientific studies have described the effect of this type of powder and the type of dyes used in them on skin and eyes, causing diseases including conjunctivitis and corneal abrasion amongst participants in these mass events [2]. In this paper, we attempt to prove that all tested Holi colors comprise more than 40% of particles whose aerodynamic diameter is below 10 μm. These are called PM10 particles (particulate matter) and can reach the lower respiratory tract if inhaled. Corn starch made up to 51% of all particles >0.7 μm and <10 μm, while particles of a size ranging between 0.7 and 10 μm ranged from 43 to 80% in the Holi colors [3].

There are no available data on the susceptibility of the colored fine powders to ignition and explosion. Ignition may be initiated, and this is due to the fact that the powders become airborne as a result of cloud formation, and become exposed to a lighter, hot surface, light bulb, the surface of a heated device, or even static electricity.

The colored powders that are used during the Holi festivals comprise certain synthetic dyes such as malachite green, auramine, methyl violet, rhodamine, and orange II, which are frequently mixed in a base material like starch or wheat flour. In order to enhance their shine, mica dust may be added to them. Corn starch or flour dust have long posed severe hazards in the food industry because of the possibility of primary explosion of airborne dust, and consequently secondary explosion. It needs to be pointed out that during the festival, the dust is spread up in the air intentionally rather than accidentally.

Unfortunately, this type of chemical is phototoxic, causes skin allergies, and is also flammable. Mica dust may cause multiple microtraumas of the skin and predispose the afflicted person to infections. In addition, the possibility of skin or ocular infections may further increase with the use of contaminated starch or wheat flour.

Holi powder, also called Holi color, Gulal powder or micro confetti, is distributed by many small companies. In most cases, the quality and scope of information provided on the external wrapping as to the ingredients of each Holi color is absolutely insufficient. To date, no European or US regulation has been adopted with respect to Holi colors, and what is more, it is not known whether they should be considered cosmetic products or general consumer goods. Therefore, the novelty of this work consists in taking into account and clarifying the flammable properties of a loose product (Holi powder) with data on the susceptibility to ignition and explosion, because flammable dusts are obligatorily subject to such tests if they are used in industry and may pose consequent risks. It is worth noting that during mass events, in case of ignition, the impact of this powder on people is disproportionately greater than in the production plant, where the requirements of occupational safety and protection, as well as explosion hazard requirements, are obligatory.

## 2. Materials and Methods

For the tests, three Holi color items were ordered from a well-known internet retailer. The supplier selection criterion was the number of completed transactions (products sold) of a popular sales broker. First of all, an analysis of the information provided on the packaging of the Holi powders acquired was carried out. All-purpose information, such as country of manufacture, ingredients, or indications applicable to health was available both on the internet and on the external wrapping of the product, and has been specified in Table 1. As corn starch is frequently used as a carrier substance for Holi colors, commercially available corn starch labelled as a food ingredient was acquired to serve as a control lot.

Dust particles smaller than 63 µm were selected as the experimental samples. An IPS particle size analyzer from Kamika Instruments (Warsaw, Poland) was used for the identification of the grain size distribution. The Sauter mean diameters of the powders were the following: Holi A powder—25.9 μm, Holi B powder—26.3 μm, Holi C powder—27.1 μm, corn starch powder—28.6 μm. Dust of such sizes can become airborne or accumulate on equipment surfaces and consequently form explosion zones. The chemical composition of Holi powder and corn starch powder was analyzed with a Vario EL Cube from Elementar Analysensysteme GmbH (Langenselbold, Germany). The chemical composition of the Holi powders analyzed is summarized in Table 2.

**Figure 1:**
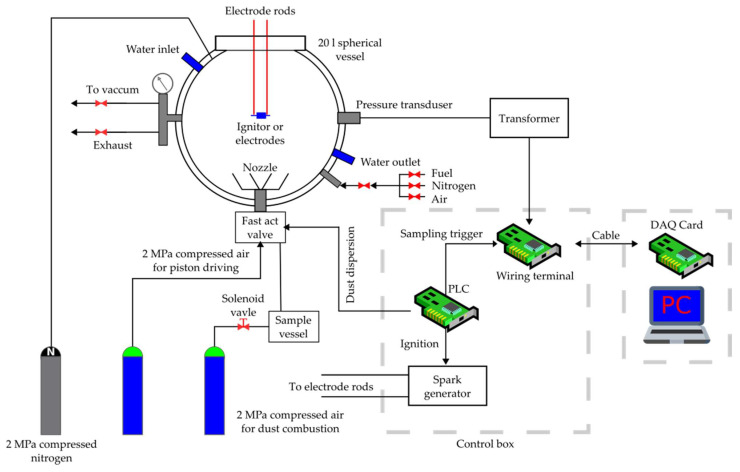
Spherical measurement device for explosion pressure Pmax, pressure increase rate (dP/dt)max, explosion index K_St_, minimum explosion concentration MEC, and decreasing oxygen concentration LOC of the Holi dust cloud.

The determination of flammable dust explosion parameters, that is to say Pmax—maximum explosion pressure, (dP/dt)max—maximum rate of explosion pressure rise, K_St—_explosion index, LEL/MEC—lower explosion limit, and LOC—limiting oxygen concentration, was carried out in a standard spherical test apparatus, 20 liters in volume, SPD-2.2 model (Figure 1), manufactured by Przedsiębiorstwo Produkcyjno Usługowe ANKO Andrzej Kołaczkowski (Warsaw, Poland). The control body was responsible for activating the powder injection sequence, triggering the ignition source, and starting the data recording system. The pressure measurement system included two pressure sensors and a recording system. The pressure measurement accuracy was up to 0.1 bar, and it included temperature influence. The time measurement accuracy amounted to 1 ms. The following experimental conditions were applied.

- Dispersion pressure (pz)—20 bar (the pressure in the dust tank after loading the test sample)
- Initial pressure (pi)—1013 mbar (the pressure in the test tank reduced to 0.4 bar so that after dust release and dispersion, the pi initial pressure value of 1013 mbar was reached)
- Initial temperature (ti)—20 °C (water cooling enabled)
- Delay time (tv)—0.6 ± 0.1 s
- Ignition source—chemical igniter with a total energy of 10 kJ (2 × 5 kJ) or 2 kJ (2 × 1 kJ).

The mixture of Holi dust with air was introduced into a vessel through a quick-acting valve and reflective nozzle. After injecting the Holi powder through the nozzle, the dust cloud was ignited by centrally mounted chemical igniters, and after a 60 ms delay a quasi-homogeneous Holi dust cloud was formed. The ignition energy of the EBBOS CHZ chemical igniter (red) from Fr. Sobbe GmbH (Dortmund, Germany) amounted to a total of 2 kJ and was used for LEL/MEC identification. The ignition energy of the EBBOS CHZ chemical igniter (green) from Fr. Sobbe GmbH (Dortmund, Germany) amounted to a total of 10 kJ and was used for Pmax and (dP/dt) max. measurement.

For each P_max_ and (dP/dt)_max_ test, three series of measurements were carried out, changing the nominal dust concentration in the 500, 750, 1000, 1250, and 1500 g/m^3^ ranges. The arithmetic mean of P_max_ and (dP/dt)_max_ measurement results obtained in each series was taken as the test result. The value (dP/dt)_max_ allows the calculation of dust explosion properties called the K_st_ value. K_st_ may be calculated based on the following formula [4]:

(1) $$K_{\mathrm{st}}={\left(\frac{\mathrm{dP}}{\mathrm{dt}}\right)}_{\max}\cdot \sqrt[{3}]{V}\;\left[\mathrm{bar}\cdot \frac{m}{s}\;\right]$$

where:

– (dP/dt)_max_ denotes the peak maximum rate of pressure increase [bar/s]
– V denotes the total internal volume of the test vessel [m^3^].

A division of explosive dusts into three classes was adopted, according to the K_st_ value (Table 3). The higher the hazard class, the greater the hazard associated with dust and air mixtures will be [5,6].

For MEC determination, three series of measurements were carried out, starting with a 500 g/m^3^ concentration, followed by 250, 125, and 60 g/m^3^.

For LOC determination, three series of (dP/dt)_max_ measurements were carried out at an oxygen concentration of 21%, in the 500 to 1500 g/m^3^ range, and if ignition was discovered, the oxygen concentration was reduced by 1%. Three series of measurements were performed for the oxygen concentration at which no ignition of the dust cloud was observed.

**Figure 2:**
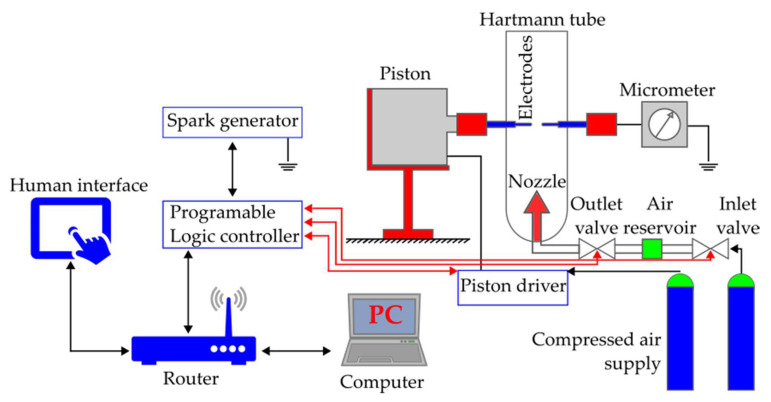
Measurement device for minimum ignition energy of Holi dust cloud.

A Hartmann Tube apparatus MINOR 2.2 from Przedsiębiorstwo Produkcyjno Usługowe ANKO Andrzej Kołaczkowski (Warsaw, Poland), as shown in Figure 2, was used for measuring the Holi dust cloud minimum ignition energy. The dusts were ignited by capacitive discharge. The apparatus consisted of a vertical glass pipe with a volume of 1.2 L, 7 cm long, including a pneumatic dust dispersing system with up to 7 bar overpressure and a spark-generating circuit. The device control panel enabled a discharge spark energy adjustment at values of 1, 3, 10, 30, 100, 300, and 1000 mJ, and setting of the discharge spark delay time from 0 to 10,000 ms (±10 ms). According to EN 13821, the ignition originated from the spreading of a self-sustaining flame from the spark discharge spot. The minimum ignition energy of the explosive dust-air mixture was the lowest value of the electric energy accumulated in the capacitor, sufficient to ignite the most flammable mixture of the dust at discharge in the pre-determined test conditions. Considering the provisions of EN 13821, the following dust concentrations were tested: 125, 250, 500, 750, 1000, 1250, 1500, 1750, 2000, 2500, and 3000 g/m^3^ at the spark circuit inductance of 1 mH and 1 µH. The tests started at the spark discharge energy level of 1000 mJ. The ignition was evaluated visually. The minimum ignition temperature was identified between the highest energy for which no ignition of the Holi dust mixture with the air occurs for ten subsequent tests and the lowest energy for which Holi dust mixture with the air ignites in any of the ten subsequent tests.

**Figure 3:**
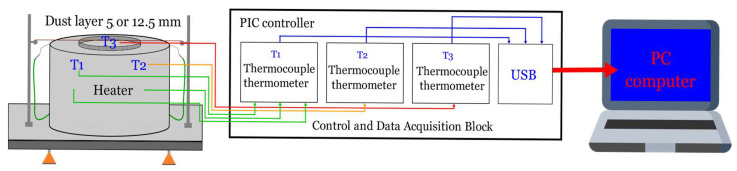
Measurement device for minimum ignition temperature of Holi dust layer.

Minimum ignition temperatures of dust layers of Holi dust were calculated to determine the minimum temperature at which the layer of dust located on a hot plate at a constant temperature undergoes thermal decomposition and/or ignites. The minimum ignition temperature of a 5 mm thick dust layer was determined at the heater plate at a constant temperature according to a standard method. The components of the LIT-3 measurement stand from Przedsiębiorstwo Produkcyjno Usługowe ANKO Andrzej Kolaczkowski (Warsaw, Poland) are shown in Figure 3.

**Figure 4:**
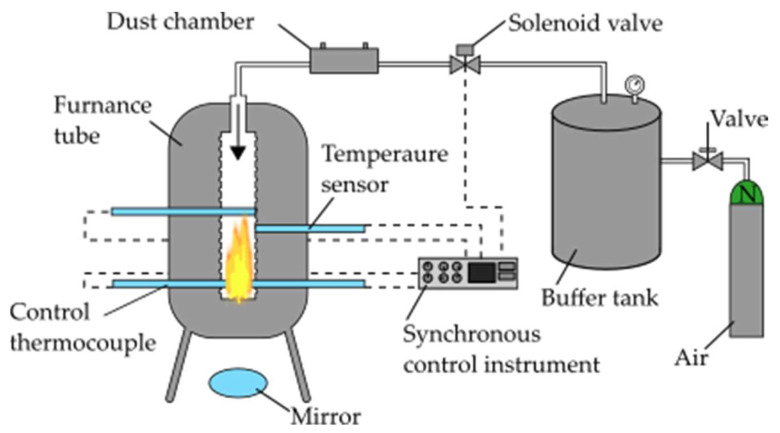
Measurement device for minimum ignition temperature of Holi dust cloud.

The minimum ignition temperature of a Holi dust cloud was determined in a MIT-3 model of a Godbert-Greenwald furnace from Przedsiębiorstwo Produkcyjno Usługowe ANKO Andrzej Kołaczkowski (Warsaw, Poland), as presented in Figure 4. A silica pipe with an open bottom 3.9 cm in diameter and 23 cm in height formed the main part of the furnace. The tests confirmed that the furnace pipe temperature was uniformly distributed. The stainless steel mirror under the pipe prevented visual monitoring of the furnace interior and the phenomena occurring inside (combustion, sparks). In each experiment, compressed air was introduced into the pipe at 10, 20, 30, and 50 kPa pressure to form a Holi dust cloud. Holi powder concentrations weighing 0.1, 0.2, 0.3, and 0.5 g were used. The first measurement was performed at 500 °C temperature, 0.3 g weight, and blown-in air pressure of 30 kPa. The weight of the Holi powder at which the flame was most intensive was determined for each case. The temperature of the heating furnace was initially set at a level liable to ignite the dust cloud, then decreased at a rate of 20 °C per step until ignition failure occurred in ten consecutive experiments.

Holi dust flammability and explosiveness in mixtures with air were tested according to the test procedures [7,8,9,10,11,12,13] described in the standards summarized in Table 4.

## 3. Results

The results of the tests conducted are shown collectively in Table 5. All tests were carried out at an ambient temperature of 20 ± 2 °C at an equal atmospheric pressure of 1000 ± 5 hPa.

**Figure 5:**
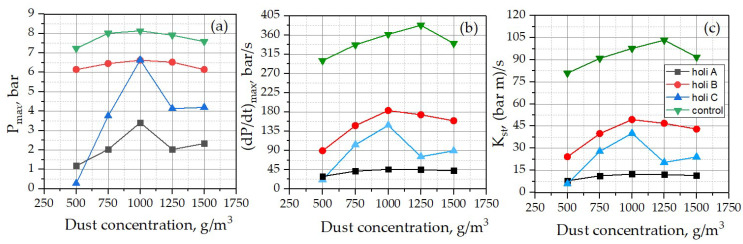
Dependence of the maximum explosion pressure (**a**), pressure increase rate (dP/dt)max (**b**) and explosion index K_St_ (**c**) on the concentration of Holi A, B, and C powders and corn starch as a reference sample.

All Holi dusts and corn starch dusts analyzed were classified as low explosive dusts (St 1). The value of the maximum pressure increase rate in the function of time for each Holi powder (dust) was recorded at the dust concentration corresponding to the maximum explosion pressure values estimated for each (Figure 5).

## 4. Discussion

It should be noted that among all Holi powders analyzed, Holi B dust was characterized by the highest value of the K_st_ parameter, amounting to 183 ± 33 m·bar·s^−1^, and the highest value of the explosion pressure increase in the function of time ((dp/dt)max), amounting to 183 ± 33 bar/s, which means that the dust may cause the most significant damage during a potential incident in open spaces, but particularly indoors.

The tests on the minimum explosive concentration revealed that the lowest concentration value at which Holi A and C dusts can form an explosive atmosphere amounts to 500 g/m^3^. The MEC values for Holi B dust and corn starch dust are lower, and Holi B dust falls within its explosiveness range already at a concentration of ca. 60 g/m^3^, while for corn starch dust, the concentration is lower and amounts to ca. 30 g/m^3^.

The LOC value is defined as the maximum oxygen concentration in the mixture of a flammable substance with air and neutral gas, at which the mixture does not explode [15]. Oxygen is the component of a flammable dust-air mixture that conditions flame propagation during fire or explosion. That is why it becomes vital to reduce the oxygen content in the mixture. Below the LOC, the dust-air mixture does not generate such a heat quantity that could enable flame propagation. Based on the tests presented, the LOC values for Holi A and C are the lowest, and amount to 20% O_2_. They are lower by 1% O_2_ than the oxygen concentration in atmospheric air, but the LOC value for Holi B dust amounts to 15% O_2_ and is only higher by two percentage points than the reference sample, corn starch dust. The oxygen content reduction to 15% O_2_ caused a decrease in the pmax value for Holi B dust of ca. 40%, whereas the (dp/dt)max value dropped by over 85% for Holi B dust with reference to the value measured in the atmosphere (21% O_2_), which suggests a strong relationship between the oxygen content and the combustion reaction kinetics.

The MIE value for a Holi dust cloud is indispensable for hazard assessment. According to the results presented in Table 5, Holi dusts are susceptible to ignition from the initials over 100 mJ, while for Holi B dust, this is over 30 mJ. An explosion of Holi B dust seems highly likely wherever dust distribution is generally non-uniform. Cases of dust ignition and explosion have occurred during Holi festivals in the past.

Holi B dust has the lowest value of dust cloud ignition temperature at 60 °C compared to corn starch dust. Holi A and Holi C dusts, even at furnace temperatures over 1000 °C, showed no dynamic combustion propagation in the entire volume of the dust-air cloud. There are three ways of influencing the dust explosiveness by the moisture content in its particles. The first involves water evaporation as a result of heating the particles. Then, water vapors mix with the pyrolysis products of organic particles, cooling and thinning them, and making the particles less reactive. The third way is to reduce the dust dispersion degree by increasing the cohesion between the particles. The moisture content in Holi powders was under 2%. Hence, the results obtained are caused by the increased dust dispersion and reduced interparticle cohesion.

The value of Holi B dust minimum ignition temperature amounted to MIT_5mm_ 430 °C ± 3.3. In contrast, for Holi A and C dusts, at the 5 mm thick layer and furnace temperature of 450 °C and at constant furnace temperature, no glowing, burning, or dust sample temperature increase of more than 250 °C above the heater plate temperature was observed, which is testimony to lack of ignition. With regard to the standard constraint that determines the temperature of the last test in the furnace as no higher than 450 °C, the value was considered to be MIT_5mm_ for both Holi dusts analyzed. Holi B dust ignited in the layer much earlier than when the heater plate temperature stabilized.

Based on the results of a dust layer minimum ignition temperature and a dust cloud minimum ignition temperature determination, the maximum temperature can be identified for system devices coated with a dust layer up to 5 mm thick or operating in the dust atmosphere. The maximum surface temperature for Holi A and C dusts should not exceed 375 °C, and it is limited mainly due to the dust deposition (the standards suggest that the MIT_5mm_ values should be reduced by 75 °C). For Holi B dust, the electric equipment temperature value should be limited to 265 °C, while for corn starch it should be limited to 306 °C. The values are conditioned by the presence of a dust cloud (the standards suggest 2/3 of Tcl values, respectively) [16,17].

The test results confirm that Holi dusts are flammable and explosive dusts, similar to their components. For instance, Table 1. of the National Fire Protection Association’s (NFPA) standard quotes corn starch dust explosion pressure at 10.3 bar and Kst of 202 m·bar·s^−1^ and hence it is classified as St1 [18]. In the online database GESTIS-DUST-EX, corn starch and talc are classified as St1.

It should be noted that an excess pressure of 0.17 causes the rupture of the tympanic membrane [19]. At an overpressure of 2 bar, there is a 99% probability of death as a result of the direct effects of explosion [18]. Powder explosion also causes heat radiation. According to Norsok Standard Z-013, at 140 °C, a human being has a five-minute tolerance limit, while at 160 °C, there is rapid unbearable pain. At 182 °C, irreversible damage occurs in 30 s, and finally, at 203 °C, the respiratory system has a tolerance level of fewer than four minutes if the skin is wet [20,21].

The significant health hazards resulting from Holi dust penetration into the skin or eye irritation should also be emphasized. In [22], an assessment of corneal penetration in a goat in a perfusion chamber containing green Holi powders tainted by malachite green was carried out. Various concentrations of this type of green, in other words 4-[(4-dimethylaminophenyl) phenyl-methyl]-N, *N*-dimethylaniline, were discovered in the color samples analyzed. One of the HPLC studies found proof of the presence of various concentrations of malachite green, which is responsible for the reported toxicity in the lavage fluid of four patients. Therefore, proof was provided that the exposure to malachite green in Holi powders causes serious eye irritation with epithelial defects.

In rats, the oral LD50 for malachite green oxalate was 275 mg/kg [23] and was ascertained to be a mutagen in the Salmonella/microsome test following metabolic stimulation. Furthermore, an examination was carried out in 40 patients who had sustained injuries as a result of contact with colors during Holi [24]. Those colors have an alkaline base and, as a result, the scale of ocular injury depends on the surface contact and the degree of penetration. The depth of the injury also depends on the degree of penetration—corneal and conjunctival epithelium, basement membrane, stromal keratocytes, stromal nerve, and the vascular endothelium. Cases of bilateral periorbital necrotizing fasciitis in a patient have also been reported [25]. A review and summary of a wide range of toxicological impacts of malachite green (triarylmethane dye) on diverse species of fish and certain mammals have been presented [26]. Furthermore, much concern has been generated over malachite green with respect to its use given its reported toxic effects. It was found that the toxicity of this dye tends to rise as exposure time, temperature, and concentration increase. Its reported consequences comprise inter alia carcinogenesis, mutagenesis, chromosomal fractures, teratogenicity, as well as respiratory toxicity.

A study was carried out on 42 patients for skin disease after the Holi festival. The most prevalent symptom was itching, as well as a feeling of burning, pain, oozing, and scaling. Symptoms experienced by the patients were thought to have been the effect of activities connected with the preparation of colors and removing those colors from their skin. The most common pattern involved eczematous lesions, as well as erosions, xerosis and scaling, erythema, urticaria, acute nail-fold inflammation, and abrasions [27,28]. Only a few color packets available on the market are provided with a warning to avoid direct contact with the skin or the mucosa, because there is not sufficient regulation or scrutiny of small manufacturing companies [29].

Holi dust poses a threat to the environment. Soil and water samples with dye-degrading bacteria added to Holi powders have been isolated and checked for bioremediation. Biological degradation of the toxicity of Holi powders in water and soil samples using Triticum vulgare showed a reduction in growth and reproduction. As they are poorly biodegradable and resistant to conventional wastewater purification processes, they are also harmful to the environment [30].

During the Holi festivities, high total suspended particulate concentrations have been recorded [31], which may also lead to adverse health effects or aggravate existing conditions, such as respiratory irritations. The use of large quantities of Holi powder during festivals causes a substantial increase in PM10 concentrations in the surrounding air. Undesirable health effects resulting from extensive exposure to high PM10 concentrations are commonly known. Cumulative concentrations of particulate matter are said to cause much higher cardiovascular and respiratory morbidity and mortality [32,33,34,35,36]. What is more, evidence has been found of the short-term effects of air pollutants and raised PM10 concentrations have also been connected to an upsurge in daily mortality [36,37]. The approximate average particle size of corn starch specified in the available literature is presumed to be 15 μm in diameter [37]. A comparison of the specific exposure in the area of the festival or its vicinity with the “normal” ambient PM10 exposure due to road traffic or combustion processes is not easy.

## 5. Conclusions

Based on the experimental tests and experiment results, the use of Holi dusts that contain corn starch and which are classified as explosive dusts is not recommended. If dusts with the flammability and explosiveness parameters mentioned in this paper were used in industry, this would entail the implementation of general fire prevention measures, such as early spark detection, automated disconnectors, general sprinkler system, and sprinklers in dusty spaces. Moreover, the essential rule of explosion protection states that the process should be carried out in a way that limits dustiness in such spaces. The size of the dust particles is of paramount importance. The European standards for the classification of explosion hazard zones provide separate definitions of dust, flammable dust, and volatile agglomerates of flammable fibers. According to [38], dust is a general term that includes flammable dusts and volatile agglomerates of flammable fibers. Flammable dust stands for fine particles of solids, with a nominal size under 500 μm, which can form explosive mixtures with the air under atmospheric pressure and at normal temperature. The dusts analyzed can be classified as flammable and explosive dusts.

The minimum particle diameter below which the combustion rate decreases depends on the duration of the three consecutive phases: release of volatile particles, gaseous phase mixing, and combustion. The dust particle diameter determines the release rate of volatile particles. This is why if the gaseous phase combustion is the slowest of the three processes, increasing the release rate of volatile particles by reducing the particle diameter will not increase the general dust combustion rate. Hence, it was demonstrated that the dust particle minimum diameter below which the combustion and explosion parameters become stable amounts to ca. 50 μm; such diameters were observed in the analyzed Holi dusts. The particle diameter for the materials whose pyrolysis gaseous products oxidate faster than the volatile dust particles is lower. A corn starch dust for which the diameter amounts to ca. 10 μm makes a good example [39]. Hence, for safety reasons, dust fineness should be limited.

Holi festivals, a characteristic feature of which is throwing large amounts of Holi powder in the air and at people, are becoming increasingly popular globally. Despite the fact that their use may cause adverse health effects, risks to environmental and fire safety, the real threat posed by Holi powders and their potential underlying mechanisms of fire and explosion hazards has not been investigated yet. The lack of regulations regarding colored Holi powders means that it is not known whether they are cosmetic or consumer products. Distributors only refer to the EN-71-3 standard, which specifies the requirements and test methods for selected materials used for making toys and their parts. Therefore, fire and explosion hazards are ignored when introducing the product.

Determining fire and explosion parameters of flammable dusts, including ways in which powders are used during mass parties, has considerable practical importance. It enables evaluation of the explosion hazard of specific technological processes that comprise the production or storage of flammable dusts [40] and proves that the same conditions as those applied during the test may occur during mass events using Holi dust.

Summing up the current situation, the minimum ignition temperature is likely to be reached in a real mass event during the Holi festival. An explosion hazard results from the presence of flammable corn starch and Holi dust, which contain very fine corn starch. Their mixture with the air may form explosive atmospheres. In the reference case, the ignition sources may include sparks caused by mechanical impact or sparks formed by friction, as well as static discharge sparks (cone, spark discharges). Unfortunately, the possibility of dust explosion during mass events where the bulk materials analyzed in the paper are used cannot be excluded. Therefore, it is worth analyzing the methods of identifying representative accident scenarios in further work [41,42].

## Figures and Tables

**Table 1:** Available information on specific Holi colors, either provided on the product itself or available online.

Type of Holi Powder	Ingredients	Declared Country of Production	Other Information	EU Regulation
Holi A (UV Holi Powder)		UK	Avoid contact with eyes. Keep out of the reach of children. If reaction occurs rinse product from affected area and discontinue use.	
Holi B (Red color powder, helloholi)	Corn starch, food grade dyes, and pigments	India	Do not use in the presence of people suffering from asthma or allergies. Avoid direct contact with eyes, do not breathe or swallow. May cause eye irritation on contact lens wearers. Do not use on irritated, damaged or sensitive skin. It can leave colored spots on bites and hair. Not intended for human consumption. Keep out of the reach of children. Store in a dark, cool and dry room. Non-toxic. It is not explosive. Does not contain heavy metals.	EN-71-3
Holi C (Pink color powder, koloryholi)	Talc, corn starch, and non-toxic pigments	India	Keep away from toddlers. Avoid contact with eyes and swallowing. Don’t use on damaged, irritated and sensitive skin. Advised to use goggles and mask. Longer celebration time.	EN-71-3

**Table 2:** Chemical composition of Holi powders.

Name	Mass [mg]	C [%]	H [%]	N [%]	S [%]
Holi A	4.973	12.41	2.029	1.38	1.018
Holi B	4.583	11.24	1.996	1.23	0.889
Holi C	3.711	20.51	1.587	1.01	0.845

**Table 3:** Division of explosive dusts into particular classes.

Hazard Class	Characteristic	K_st_ [m·bar/s]
St 1	Weak to moderate explosion	<200
St 2	Strong explosion	>200 and <300
St 3	Very strong explosion	>300

**Table 4:** Standard procedure used in the study.

Parameter	Standard
P_max_	EN 14034-1
(*dP*/*dt*)_max_	EN 14034-2
*K*_st_	EN 14034-2
*MEC*	EN 14034-3, ISO/IEC 80079-20-2
*LOC*	EN 14034-4
*MIT*_5mm_	EN 50281-2-1, ISO/IEC 80079-20-2
*T*_cl_	EN 50281-2-1, ISO/IEC 80079-20-2
*MIE*	EN 13821, ISO/IEC 80079-20-2

**Table 5:** Comparison of flammability and explosiveness of the Holi and corn starch dusts analyzed [7,8,9,10,11,12,13,14].

Parameter	Unit	Holi A	Holi B	Holi C	Corn Starch (Control Sample)
P_max_	bar	3.4 ± 0.3	6.6 ± 0.3	6.7 ± 0.3	8.2 ± 0.3
(*dP*/*dt*)_max_	bar/s	46 ± 23	183 ± 33	149 ± 25	383 ± 32
*K*_st_	(m·bar)/s	12 ± 13	50 ± 9	40 ± 10	103 ± 11
*St*	–	St1	St1	St1	St1
*MEC*	g/m^3^	500 ± 11.5	60 ± 9.8	500 ± 12	30 ± 9.2
*LOC*	%O_2_	20 ± 1	15 ± 1	20 ± 1	13 ± 1
*MIT*_5mm_	°C	450 ± 2.9	430 ± 3.3	450 ± 3.3	420 ± 3.2
*T*_cl_	°C	>1000	400 ± 3.8	>1000	460 ± 3.6
*MIE*	mJ	100 < MIE < 300	30 < MIE < 100	100 < MIE < 300	30 < MIE < 100
